# Isolation and Characterization of Atrazine Mineralizing *Bacillus subtilis* Strain HB-6

**DOI:** 10.1371/journal.pone.0107270

**Published:** 2014-09-19

**Authors:** Jinhua Wang, Lusheng Zhu, Qi Wang, Jun Wang, Hui Xie

**Affiliations:** 1 College of Resources and Environment, Key Laboratory of Agricultural Environment in Universities of Shandong, Shandong Agriculture University, People's Republic of China; 2 Ministry of Education Key Laboratory of Pollution Processes and Environmental Criteria, Nankai University, People's Republic of China; Loyola University Medical Center, United States of America

## Abstract

Atrazine is a widely used herbicide with great environmental concern due to its high potential to contaminate soil and waters. An atrazine-degrading bacterial strain HB-6 was isolated from industrial wastewater and the 16S rRNA gene sequencing identified HB-6 as a *Bacillus subtilis*. PCR assays indicated that HB-6 contained atrazine-degrading genes *trz*N, *atz*B and *atz*C. The strain HB-6 was capable of utilizing atrazine and cyanuric acid as a sole nitrogen source for growth and even cleaved the *s*-triazine ring and mineralized atrazine. The strain demonstrated a very high efficiency of atrazine biodegradation with a broad optimum pH and temperature ranges and could be enhanced by cooperating with other bacteria, suggesting its huge potential for remediation of atrazine-contaminated sites. To our knowledge, there are few *Bacillus subtilis* strains reported that can mineralize atrazine, therefore, the present work might provide some new insights on atrazine remediation.

## Introduction

Atrazine (2-chloro-4-ethylamino-6-isopropylamino-1,3,5-triazine), a popular herbicide, is used world-wide to inhibit the growth of unwanted broad-leaf plants in agriculture [1]. Because it is moderately persistent under normal soil conditions and has low to moderate water solubility, atrazine and its derivatives have even been detected in soils, surface and ground waters [2], [3]. Moreover, atrazine has been proposed as a possible carcinogen, endocrine disrupter and teratogen, affecting ecosystems and human health [4], [5]. An increasing concern about the wide contamination and toxicological properties of atrazine has prompted researchers to seek bioremediation options for atrazine removal [6], [7].

Microbial degradation has been regarded as the most important mechanism of atrazine degradation in contaminated sites. Up to date, a number of microorganisms with different atrazine degradation efficiencies and growth characteristics are reported. Some of these strains can only metabolize atrazine to cyanuric acid [2], [8], while others can break the *s*-triazine ring and mineralize atrazine [9], [10]. These studies show wide variation of the ability of bacteria to degrade atrazine, however, the degradation efficiencies of the bacteria are not very high in most cases. Our previous work shows that a bacterial strain (HB-5) is capable of utilizing atrazine as a sole carbon and nitrogen source and exhibits faster atrazine degradation rates in atrazine-containing mineral media than the well-characterized atrazine-degrading bacteria *Pseudomonas sp.* ADP [2]. Unfortunately, the strain HB-5 can only transform atrazine, but not mineralize atrazine. Therefore, it is necessary to isolate highly effective bacteria, especially atrazine-mineralizing bacteria for atrazine-contaminated sites remediation.

It has been postulated that bacteria consortia appear to be more common and more efficient than individual species and mixed cultures may be more capable of degrading a given pollutant completely [11], [12]. So far, some consortia are reported to be more efficient in their cooperative catabolic pathways than the role of individual members within different atrazine-degrading communities, but synergistic effect between consortium members has been less reported [12], [13]. The characterization of atrazine-degrading bacterial strains revealed the existence of *atz*A, B, C, D, E and F genes encoding enzymes responsible for the mineralization of this herbicide [14], [15]. Two other atrazine-degrading genes, *trz*N gene coding an atrazine chlorohydrolase and the *trz*D gene coding a cyanuric acid hydrolase, have also been studied [16], [17]. The *atz*A/*trz*N, *atz*B, and *atz*C genes encode the enzymes to catalyze the sequential hydrolytic reactions transforming atrazine to cyanuric acid [18], [19]. Cyanuric acid is then further hydrolyzed to carbon dioxide and ammonia catalyzed by the enzymes encoded by the *trz*D/*atz*D, *atz*E and *atz*F genes [15], [20].

In the present study, a high-efficiency atrazine degrader, *Bacillus subtilis* strain (HB-6), was isolated from industrial wastewater. The objective of this study was to characterize ability of HB-6 to degrade atrazine, the genes involved in atrazine degradation, and the cooperative relation among the mixed cultures in accelerating atrazine degradation.

## Materials and Methods

### Chemicals and Media

Atrazine (99.9% purity) was purchased from AccuStandard, Inc., USA. Hydroxyatrazine (2-hydroxy-4-ethylamino-6-isopropylamino-1,3,5-s-triazine) (99.0% purity) and cyanuric acid (1,3,5-striazine-2,4,6-triol) (99.0% purity) were purchased from J &K Chemical Ltd, Beijing, China. Urea (99.9% purity) was purchased from National Institute of Metrology, Beijing, China.

Enrichment medium consisted of a mineral salts medium (MSM) and 100 mg·L^−1^ atrazine as the sole nitrogen source, plus sucrose 3 g·L^−1^ as carbon source, and was autoclaved at 121°C for 30 minutes. The mineral salts medium (MSM) contained (per liter) 1.6 g of K_2_HPO_4_, 0.40 g of KH_2_PO_4_, 0.20 g of MgSO_4_·7H_2_O and 0.10 g of NaCl. Trace element solution contained (per liter): EDTA, 2.5 g; FeSO_4_·7H_2_O, 1.0 g; ZnSO_4_·7H_2_O, 5.0 g; MnSO_4_·H_2_O, 1.0 g; CuSO_4_·5H_2_O, 0.40 g; Na_2_B_4_O_7_·10 H_2_O, 0.20 g; Na_2_M_O_O_4_·2H_2_O, 0.25 g. The pH of the medium was adjusted to 7.0 with 1 M NaOH solution.

The isolated microorganisms were maintained in agar slants consisting of the MSM supplemented with 16 g of agar per liter complemented with cyanuric acid or atrazine, 100 mg·L^−1^ plus sucrose, 3 g·L^−1^.

### Enrichment and Isolation of Atrazine-degrading Bacteria

Enrichment and isolation method was performed according to Wang et al. (2011) [2]. Wastewater samples obtained from an atrazine production plant were used to inoculate enrichment cultures. Five milliliters of the wastewater sample was added to a 250-mL flask with 100 mL enrichment medium. It was incubated aerobically with shaking at 160 rpm at 30°C for 7 days. Aliquots were subcultured every 3 days for total of four passages to obtain a mixed culture. The final culture was diluted and plated on agar plates containing atrazine (100 mg·L^−1^). Mature colonies were repeatedly streaked on agar plates for isolation of a pure culture. A bacterial isolate, designated strain HB-6, showed high atrazine biodegradation activity and was selected for further analysis.

### Identification of Stain HB-6

Identification of the isolates was performed by conventional methods based on biochemical reactions and confirmed by 16S rDNA sequencing. Total DNA was extracted from HB-6 by conventional phenolic extraction and isopropyl alcohol precipitation. The primer pair of 27F (5′-AGAGTTTGATCMTGGCTCAG-3′) and 1492R (5′-CGGYTACCTTGTTACGACTT-3′) were used for amplification of 16S rDNA of HB-6, and the product was approximately 1600 bp. The PCR cycle parameters were as follows: pre-heating at 94°C for 5 min, 32 cycles of denaturation at 94°C for 1 min, annealing at 57°C for 1 min and extension at 72°C for 2 min, and a final extension for 10 min at 72°C. After purification by agarose gel electrophoresis, PCR fragments were ligated to the linearized vector pMD20-T (TaKaRa Biotechnology Co. Ltd., China), and transformed into competent *Escherichia coli* DH5α cells. Sequencing of the cloned insert was performed by Shanghai Invitrogen Technology Co. Ltd., China. The DNA sequences were analyzed using the BLAST program of the National Centre for Biotechnology. DNA sequences of related bacteria and some known type strains were used to construct a phylogenetic tree using MEGA4.

### Mineralization of Atrazine and Cyanuric Acid by HB-6

Strain HB-6 was incubated in MSM with 100 mg·L^−1^ of atrazine as nitrogen source, plus sucrose 3 g·L^−1^ as carbon source. Cells grown in atrazine liquid medium were harvested by centrifugation at 8000×g for 30 min, washed twice with phosphate buffer (pH 7.2), and resuspended in fresh atrazine liquid medium to an optical density at 600 nm of 0.2, and then inoculated into MSM by transferring 5% (v/v) culture volume. Degradation was conducted in 250-mL flasks containing 100 mL MSM with 100 mg·L^−1^ of atrazine as the sole nitrogen source. The culture was cultivated at 30°C and aerated at 160 rpm. All analysis was carried out in three biological replicates.

To confirm that HB-6 can degrade cyanuric acid during the total breakdown of atrazine, cyanuric acid was also tested as an initial substrate. HB-6 cells were prepared and inoculated in the same manner as in the atrazine-degrading experiment. All analysis was carried out in three biological replicates.

Furthermore, the strain HB-5 obtained in our previous work [2] and strain HB-6 were incubated together in MSM with atrazine as a nitrogen source in order to improve the degradation rate of atrazine. The cells were prepared and inoculated in the same manner as in the atrazine-degrading experiment. All analysis was carried out in three biological replicates.

Atrazine was quantified by HPLC (Aglient 1100) equipped with a variable-wavelength UV detector and fitted with a reverse-phase C18 column (Eclipse XDB-C18; size: 250 mm×4.6 mm (i.d); particle size: 5 µm). Mass spectrometry was used to determine the mass of the metabolites. The Agilent HPLC/MSD as equipped with an electrospray ionization source (ESI), an ion trap mass analyzer with a capillaryvoltage of 4000 V, nebuliser gas (N_2_ temperature of 350°C) of 30 V and the ion trap scanned from 50 to 1000 *m/z*, the HPLC system described above served as the inlet. Authentic standards of atrazine and putative degradation intermediates were used.

For atrazine detection, the flow rate was 0.8 mL·min^−1^ (methanol/water = 70∶30, v:v), the variable-wavelength UV detector was set to 222 nm. For hydroxyatrazine detection, the flow rate was 1.0 mL·min^−1^ (methanol/water = 80∶20, v∶v), the variable-wavelength UV detector was set to 243 nm. For cyanuric acid detection, the flow rate was 0.7 mL·min^−1^ (methanol/water = 40∶60, v∶v), the variable-wavelength UV detector was set to 216 nm. All of the injection volumes were 5 µL.

### PCR Assays of Atrazine-degrading Genes

Total genomic DNA was extracted and used as the PCR template. The atrazine-degrading *atzA*, *atzB*, *atzC*, *atzD*, *atzE and atzF*, *trzN* genes of strain HB-6 were detected by PCR with the primers and conditions previously reported [6], [14], [16], [18]. PCR reaction was performed with the following thermocycle program: 94°C for 5 min, 32 cycles of 94°C for 1 min, X°C for 1 min, 72°C for 2 min, and a final extension for 10 min at 72°C. The resulting sequences were compared with the genes available in the GenBank nucleotide library by a BLAST search through the National Center for Biotechnology Information (NCBI) Internet site.

## Results and Discussion

### Isolation and Identification of Atrazine-degrading Bacteria

Among the selected isolates, one strain designated as HB-6 was selected for further study due to its high degradation activity on atrazine. Strain HB-6 produced obvious clearing zones around the colonies on agar medium amended with atrazine (1000 mg·L^−1^). This phenomenon indicated the ability of the bacterium to metabolize very high concentrations of atrazine in a solid matrix [21]. Furthermore, HB-6 was capable of utilizing atrazine or cyanuric acid as a sole nitrogen source for growth. In fact, energy required for bacterial growth was not adequate from such toxic compounds, either atrazine or cyanuric acid, so we added sucrose 3 g·L^−1^ as carbon source. In such cases, addition of easily biodegradable organic compounds stimulated the growth of the bacteria, and thereby enhanced the degradation rate by utilizing recalcitrant compound as secondary substrate in co-metabolic process [22].

**Figure 1:**
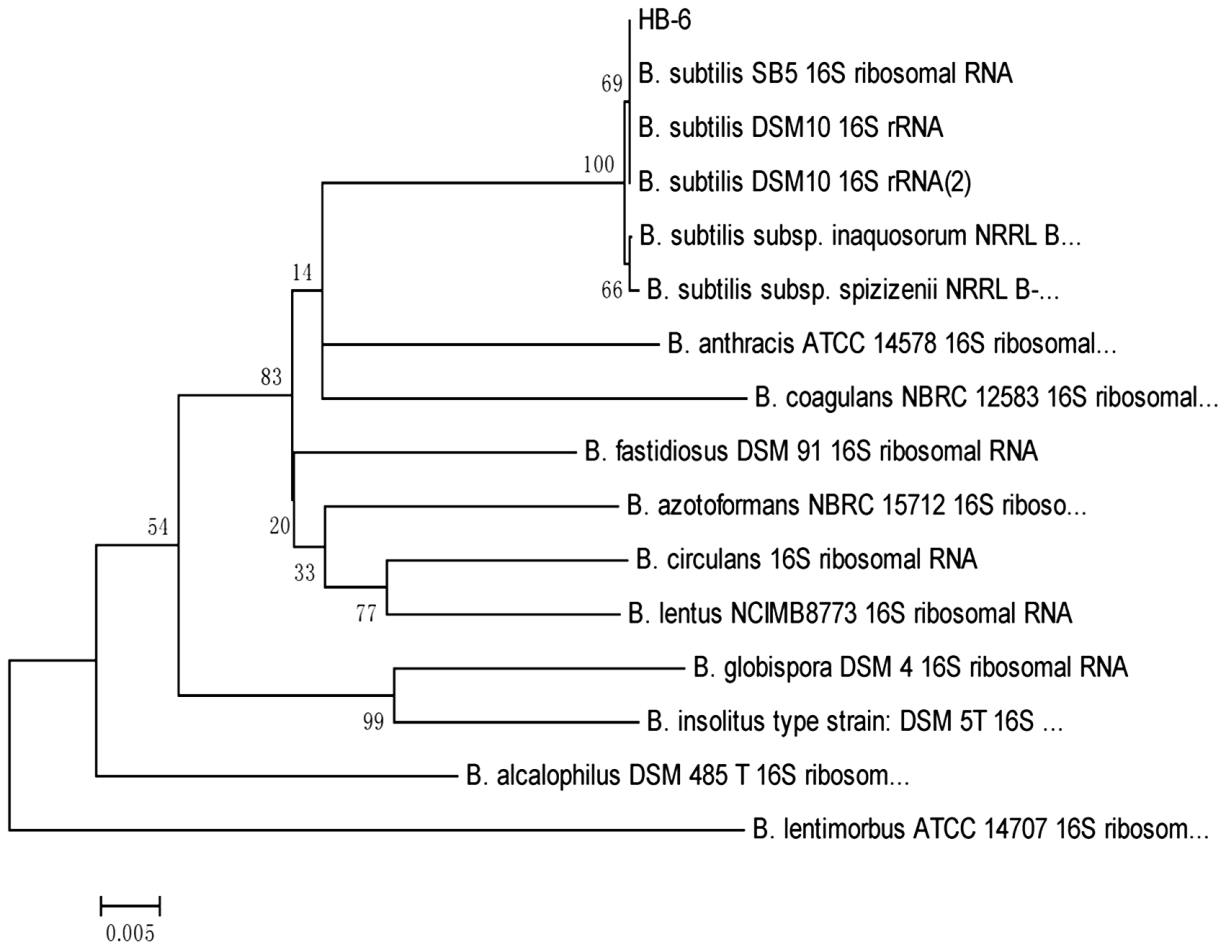
Phylogenetic tree based on the 16S rDNA sequence data. Numbers at the nodes indicate bootstrap values from the neighborhood-joining analysis of 1,000 resampled data sets. The bar indicates 0.005 substitution per nucleotide position.

Morphological and physiological characterization indicated that HB-6 was a Gram-positive yellow-pigmented bacterium. The 16S rRNA sequence of strain HB-6 (1,441 nucleotides) was compared to those of the bacterial sequences in GenBank. Strain HB-6 exhibited a high sequence similarity with bacteria classified as *Bacillus subtilis* and the highest sequence similarity (100%) was found with *Bacillus subtilis* (Figure 1). The accession number of the 16S rRNA gene sequence in GenBank was HM116874. According to its morphology, physiologic and biochemical characteristics mentioned previously, together with the phylogenetic analysis, the strain HB-6 was identified as a *Bacillus subtilis*. Previous studies reported that members of genus *Bacillus subtilis* have been linked to biodegradation of many environmental contaminants such as triphenylmethane [23], p-aminoazobenzene [24], di-2-ethylhexyl phthalate [25], benzo[a]pyrene [26], and benzene [27]. These works have shown the strong potential of *Bacillus subtilis* for bioremediation of contaminated sites.

### Degradation Characteristics of Atrazine by HB-6

**Figure 2:**
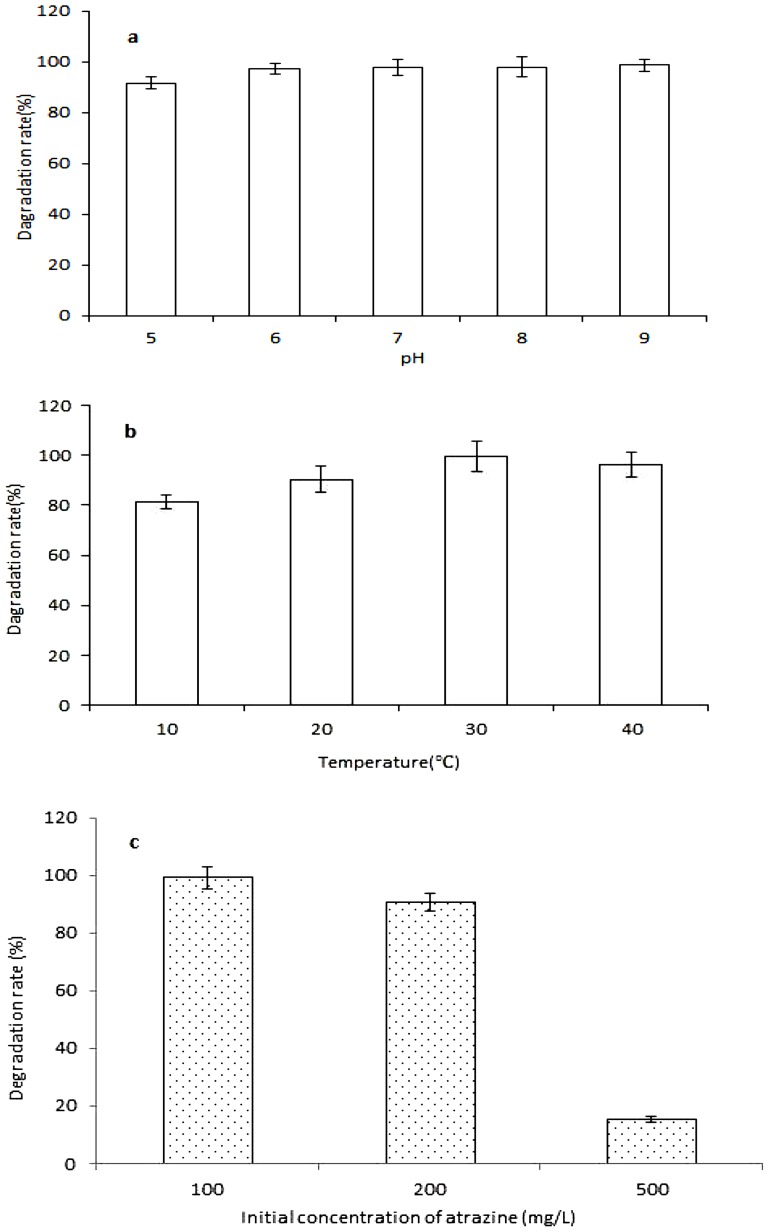
Degradation of atrazine by strain HB-6 at different culture conditions. Different pH values (a), temperatures (b) and initial concentration of atrazine (c). Error bars represent the standard deviation of three replicates.

Figure 2 shows biodegradation of atrazine at different pH values, culture temperatures and initial concentration after 24 h incubation. Under the incubation pH and temperature conditions, the strain HB-6 could degrade atrazine rapidly and the degradation rate of atrazine (initial concentration was 100 mg·L^−1^) remained over 80%. The results of the present study demonstrate that isolate HB-6 can efficiently degrade atrazine and the optimal temperature and pH ranges for atrazine biodegradation were wide, indicating that the organism has strong ability to survive and metabolize atrazine in contaminated environments.

Atrazine (100 mg·L^−1^) was almost completely removed from the medium by HB-6 after 24 h of incubation, and a nearly 90% and 20% removal rates were observed even when the initial atrazine concentrations were as high as 200 mg·L^−1^ and 500 mg·L^−1^, respectively (Figure 2c). Compared with other atrazine-degrading microorganisms, the isolate HB-6 had stronger atrazine-degrading ability, and the biodegradation efficiency was 7.5 mg·L^−1^·h^−1^ (Table 1 [2], [3], [21], [28]–[33]). These results illustrate the high bioremediation potential of *Bacillus subtilis* for heavily contaminated sites.

**Table 1:** Comparison of degradation efficiency of atrazine-degrading microorganisms.

Strain	Initial concentration	Time	Degradation rate	Efficiency	Reference
	(mg·L^−1^)	(h)	(%)	(mg·L^−1^·h^−1^)	
HB-6	200	24	90	7.50	This study
B-30	16	72	100	0.22	[28]
ADP	100	24	100	4.17	[21]
NI86/21	55	48	100	1.15	[29]
J14a	50	72	94	0.65	[30]
AD1	300	48	100	6.25	[31]
MCM B-436	25	30	100	0.83	[32]
AD26	300	72	95	3.96	[33]
HB-5	100	18	100	5.56	[2]
DAT1	500	48	95	10.42	[3]

**Figure 3:**
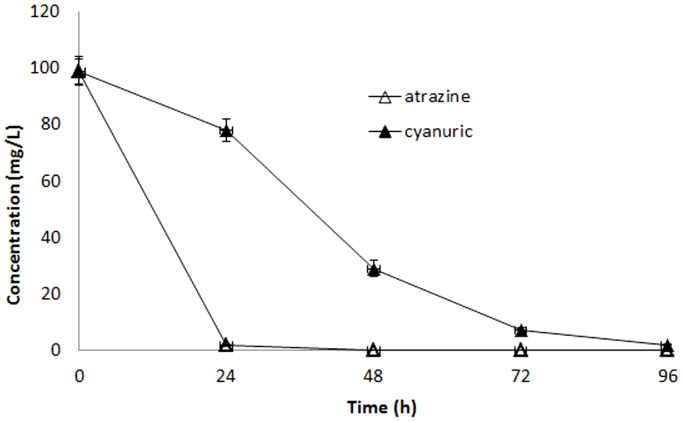
Degradation of atrazine and cyanuric acid by HB-6. △, atrazine as the initial compound in the medium; ▴, cyanuric acid as the initial compound in the medium. Error bars represent the standard deviation of three replicates.

Our previous study [2] shows that strain HB-5 can only transform atrazine to cyanuric acid, a known atrazine intermediary metabolite, but can’t mineralize atrazine. Therefore, high-efficiency atrazine-degrading bacteria that can degrade cyanuric acid and then mineralize atrazine are necessary to be isolated. In order to confirm that HB-6 could degrade cyanuric acid, cyanuric acid was supplied as the initial substrate in the MSM (Figure 3). Figure 3 shows the degradation of atrazine and cyanuric acid by HB-6. Atrazine was almost completely removed from the medium after 24 h of incubation, while cyanuric acid was degraded over 90% (concentration from 100 mg·L^−1^ to 7.7 mg·L^−1^) after 72 h of incubation.

Numerous atrazine-degrading organisms can only transform atrazine to cyanuric acid but can’t mineralize atrazine [2], [18], [34]. Previous works have shown that cyanuric acid has negative effect on environment and organisms [35]. Therefore, cyanuric acid-degrading bacteria are necessary to be isolated. In the present study, the strain HB-6 could degrade cyanuric acid rapidly. Cyanuric acid could replace atrazine as the sole source of N, indicating that the organism was capable of ring cleavage. It is reported that the *Pseudomonas sp.* strain D can grow with cyanuric acid as sole source of nitrogen and cyanuric acid is entirely metabolized concomitantly with growth [36]. Atrazine-degrading bacteria strain M91-3 [37], strain NRRLB-12227 [17], and strain ADP [9], are reported also capable to degrade cyanuric acid quickly.

### Mineralization of Atrazine and Cyanuric Acid by HB-6

**Figure 4:**
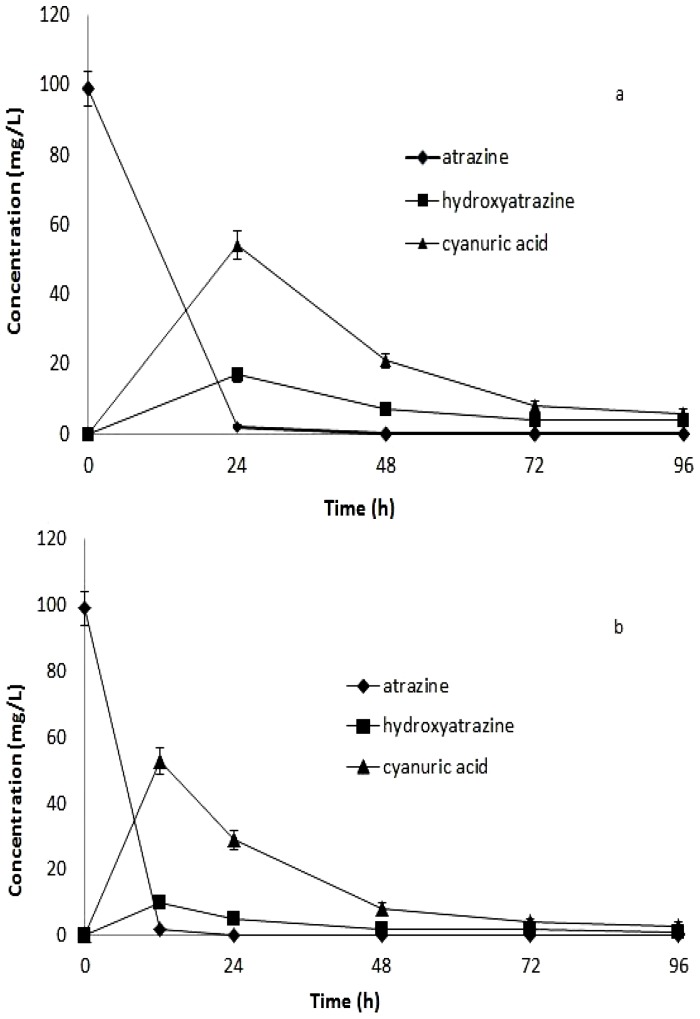
HPLC of extracts from cell supernatants after metabolism of atrazine by HB-6 or HB-6+HB-5. Degradation by HB-6 (a), degradation by HB-6 together with HB-5 (b). Error bars represent the standard deviation of three replicates.

Earlier studies report that hydroxyatrazine and cyanuric acid are the two major metabolites of atrazine by bacteria. Samples collected from the growth media were subjected to HPLC analysis. Figure 4a shows the change of atrazine and two main metabolites. The amount of atrazine decreased rapidly, and hydroxyatrazine was observed to accumulate transiently and then undergo gradual degradation accompanied by the accumulation of cyanuric acid. Meanwhile, cyanuric acid reached its peak concentration after 24 h mineralization, and then decreased quickly. It was consistent with literature that atrazine can be transformed to hydroxyatrazine and cyanuric acid, then the two metabolites show an obvious decreasing trend [10].

Our previous study indicates that strain HB-5 with a very high atrazine degradation rate can transform most of the atrazine in liquid medium within 12 h. Therefore, strain HB-6 and HB-5 were incubated together in MSM with atrazine as a nitrogen source in order to improve the degradation rate of atrazine (Figure 4b). During the whole course of the experiment, changes in the three chemicals in medium with both HB-6 and HB-5 were similar with that in medium with HB-6, while the degradation of all the chemicals became faster in medium with HB-5. The results indicated that both HB-6 and HB-5 could co-exist and work together well.

It has been postulated that mixed cultures are likely to have a greater capacity to degrade substrates by virtue of increased catabolic capability and the rates of growth and substrate utilization are frequently higher in enriched mixed cultures than those rates in pure cultures isolated from the mixture [12]. For atrazine degradation, the metabolism might require the combined metabolic activities of more than one organism and some consortia are reported for their metabolic cooperative actions in atrazine degradation [12], [13], [38]. Some reports have showed that the atrazine-degrading pathway may be operating in different atrazine-degrading bacterial communities. An example of cooperative metabolism occurs within a complex eight-member atrazine-degrading consortium with two pathways of atrazine degradation [13]. In the present study, the strain HB-5 and HB-6 had different atrazine-degrading pathways, but they could work well together and both of the organisms could play their role in atrazine degradation. A stable four-member bacterial consortium, DNC5 that is capable of metabolizing atrazine also shows the synergistic effect between consortium members [12].

**Figure 5:**
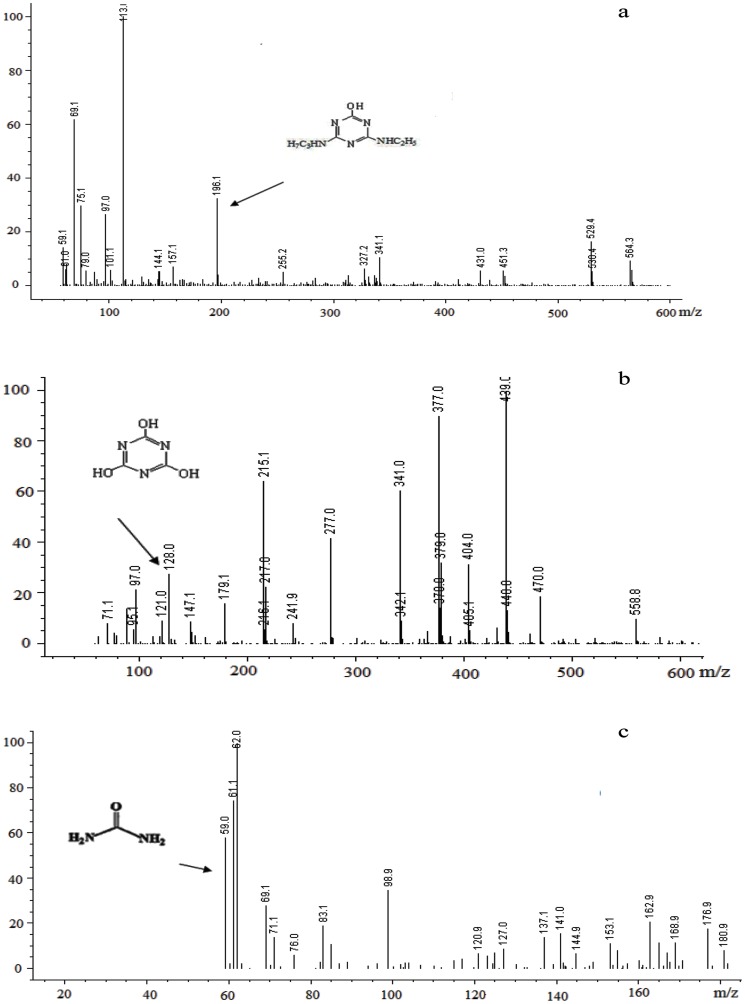
HPLC-MS identification of the metabolites produced from atrazine by HB-6. Hydroxyatrazine (a), cyanuric acid (b) and urea (c).

**Figure 6:**
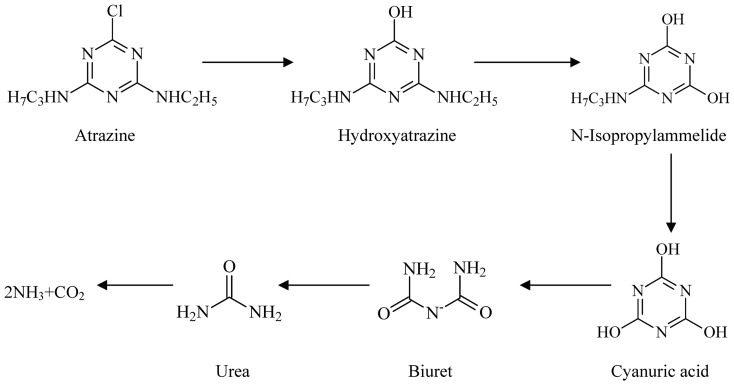
Proposed pathway for degradation of atrazine by strain HB-6.

To identify the major metabolites of atrazine by HB-6, HPLC–MS was used to confirm the putative metabolites and determine whether they are true pathway intermediates of atrazine. A metabolite with *m/z* (M-1) of 196.1 (Figure 5a) was identified as hydroxyatrazine, the metabolite with *m/z* (M -1) of 128.0 (Figure 5b) was identified as cyanuric acid, and the other metabolite with *m/z* (M -1) of 59.0 was identified as urea, respectively. There was no other metabolite(hydroxyatrazine and cyanuric acid)accumulation except urea in the MSM when the culture medium after metabolism of atrazine by HB-6 was analyzed. Therefore, the proposed pathway for degradation of atrazine by strain HB-6 was shown in Figure 6, indicating that HB-6 could mineralize atrazine.

Some reports have been published that the atrazine-degrading pathway may be from cyanuric acid to biuret, urea, carbon dioxide and ammonia [15], [37]. Cyanuric acid is previously thought to be hydrolyzed to urea, but some recent studies have shown that the bacteria studied produce biuret to generate allophanic acid as an intermediate [12]. In the present study, on the basis of the mineralization data and the utilization of cyanuric acid as an N source, it was clear that the isolate HB-6 might cleave the s-triazine ring and thereby produce urea, carbon dioxide and ammonia. Most commonly, the bacterial metabolism of atrazine is reported to occur via enzymatic, hydrolytic displacement of substituents from the three carbon atoms of the *s*-triazine ring. The broad-specificity enzymes TrzN/AtzA, AtzB, and AtzC can funnel dozens of s-triazine ring compounds into cyanuric acid, the enzyme AtzD/TrzD acts in concert with AtzE and AtzF to hydrolyze cyanuric acid to yield 3 moles each of carbon dioxide and ammonia [9], [15], [39]. Therefore, further work has to focus on the set of enzymes associated with atrazine degradation.

### Atrazine-degrading Genes in HB-6

**Figure 7:**
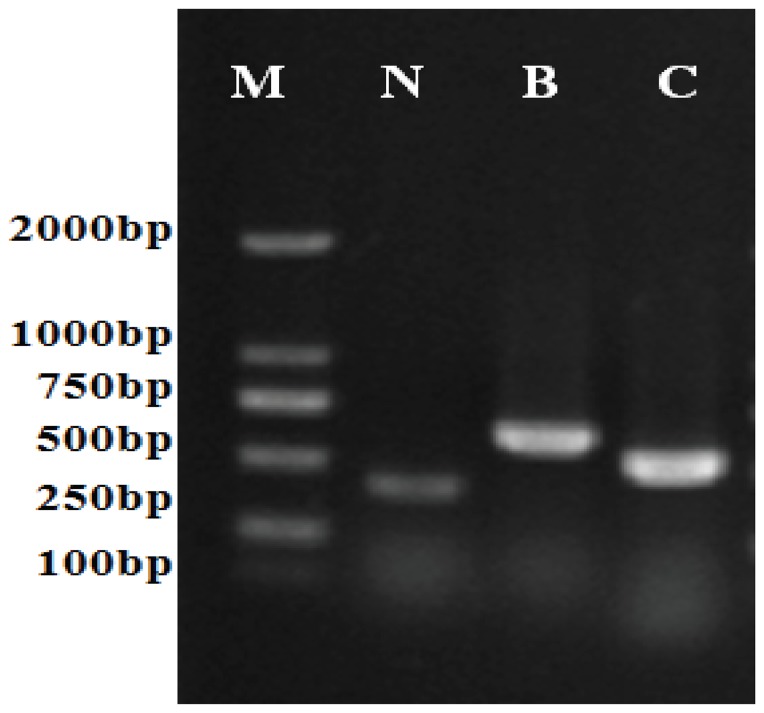
Atrazine-degrading gene amplification of HB-6. Lane 1: DNA marker, lane 2: *trzN* of HB-6, lane 3: *atzB* of HB-6, lane 4: *atzC* of HB-6.

We amplified the genes encoding the atrazine- degrading enzymes t*rzN*, *atzA*, *atzB*, *atzC*, *atzD*, *atzE* and *atzF*, using the primers reported and the expected 0.4-kb *trzN* 0.6-kb *trzB* and 0.5-kb *atzC* PCR products were obtained (Figure 7). The *trzN* PCR product was 99% similar to the *trzN* gene of *Arthrobacter aurescens* strain TC1, and the atzB and *atzC* PCR products were 99% identical to the a*tzB* and atzC gene of *Pseudomonas sp.* strain ADP. Attempts to detect the previously reported gene *(atzA*) or genes encoding enzymes involved in cyanuric acid degradation (*atzD–atzE–atzF*) were unsuccessful.

The *atzA, atzB* and *atzC* genes, which encode the activities required for removal of the chlorine and aminoalkyl side chains of atrazine to yield cyanuric acid, are found in the best-characterized atrazine-degrading strain ADP [14]. The *atzA*, *atzB* and *atzC* genes are considered to be globally distributed and be highly conserved in diverse genera of bacteria [14], [31], [40]. Moreover, a gene encoding a chlorohydrolase, *trz*N, that carries out the same function as *atz*A is characterized in *Nocardioides sp.* strain C190 [16]. Since its discovery, a number of organisms have been isolated that contain *trz*N rather than *atz*A, and the *trzN* is reported to have broader substrate specificity than *atzA* [19], [41]–[43]. We successfully detected the *trzN, atzB* and *atzC* genes in HB-6, which meant the three genes encoded the relevant degrading enzymes to transform atrazine to cyanuric acid. The *atzD*, *atzE* and *atzF* genes which can encode enzymes to mineralize cyanuric acid to carbon dioxide and ammonia [15], [44] failed to be detected in HB-6. This result indicated that there might be some other genes in strain HB-6 that assisted in the degradation of cyanuric acid. Therefore, further research is needed to study the potential genes in strain HB-6 to understand the molecular bases involved in the mineralization of cyanuric acid.
